# A Contribution to the Study of the Structure of the Dentine,—the So-Called “Sheaths of Neumann”

**Published:** 1895-11

**Authors:** R. R. Andrews


### International Dental Journal.

Vol. XVI. November, 1895. No. 11.

### Original Communications.[1]

### A CONTRIBUTION TO THE STUDY OF THE STRUCTURE OF THE DENTINE,—THE SO-CALLED "SHEATHS OF NEUMANN."[2]

BY R. R. ANDREWS, A.M., D.D.S., F.R.M.S.

If we place a tooth in strong acid, within a few days it will become wholly decalcified, and there will remain only a transparent, slimy, jelly-like mass. If a portion of this be transferred to a glass slide, covered and examined under high powers of the microscope, it will be found to consist almost wholly of tangled tubes, looking like threads, crossing the field of view in every direction. These thread-like bodies are the so-called "sheaths of Neumann." We shall find among them smaller tubes of different lengths, which are probably the linings of the fibres of the cement, —the fibres of Sharpey. We shall also see small, irregular-shaped bodies, and these are the lining of the cement-corpuscles, the lacunæ of the cement. If it be a partially-formed tooth which we decalcify, we shall also find a narrow layer, which is between the formative cells and the formed dentine. In regard to these long,

[1] The editor and publishers are not responsible for the views of authors of papers published in this department, nor for any claim to novelty, or otherwise, that may be made by them. No papers will be received for this department that have appeared in any other journal published in the country.

[2] Read before the American Academy of Dental Science, Boston, February 6, 1895.

thread-like tubes, which we shall see later on the screen, Tomes makes the statement that they demonstrate that the tubes of the dentine have definite walls, and this is the subject-matter we are to discuss later. It demonstrates the fact that they are composed of a substance singularly indestructible, and places them, on this account, with the tissues that are found on the border-land of calcification,—a tissue composed of calcoglobulin which has been deposited in a fibrous, gelatin-like substance, first formed when a tissue is to become calcified.

I shall ask your attention to a brief view of what has been written about the tubular structure of the dentine. It is stated that Leuwenhoek, a brilliant investigator of nearly two hundred years ago, who made his many and valuable discoveries by the aid of his simple microscope, was really the first to discover the structure of the dentine. He described it as composed of little tubes. About a hundred and sixty years afterwards the subject created considerable discussion,—thus Monro believed the teeth were longitudinally fibrous, and Fox that they were deposited in layers; Cuvier, that they were analogous to rock and fibrous; Serres says that there is nothing like fibres in them; Rousseau describes them as being longitudinally striated; and Blanding, who wrote as late as 1836, tells us that they are composed of plates situated parallel to their external surfaces. It remained for Retzius to discover and describe that there were minute tubes in dental bone, "and these are a peculiar kind of vessel, containing a nourishing and supporting fluid." Later in his work he contradicts this, saying, "the small osseous tubes contain only osseous earth." This is somewhat conflicting. How a tooth can be filled with osseous earth and yet allow the circulation of a nourishing and supporting fluid is in itself somewhat puzzling.

Recently, while reading Nasmyth's work on "Researches on the Development, Structure, and Diseases of the Teeth," I was somewhat interested in reading of a very eminent naturalist, who stated that a curious and interesting natural phenomenon took place. He made this statement: that the mouths of the vessels (tubes) which have been cut in the operation of stopping (filling a tooth) deposit a layer of calcareous matter through the tubes to the cut surface under the stopping (filling). This statement was made about 1838. And the late Robert Arthur, of Baltimore, held to this same theory. He says, in the teeth of our younger patients, the dentine possesses a power not generally known, of making an effort to protect itself from destruction. In a cut or broken surface which is kept clean, the tubes exposed become filled up with a dense, ivory-like substance, and this surface seldom decays afterwards. Tomes tells us that when we are examining a cross-section of the dentinal tubes, there will be seen around the opening of the canal, when examined by a high power, a thin yellowish border, which, he says, may be the sheath of Neumann; but, somewhat uncertain, he continues, it must be remembered that the dentinal sheaths can only be fully demonstrated by processes which amount to a partial destruction of the dentine, and that therefore they are in some degree, at all events, artificial. And it may be that they have no real existence until they are brought into existence by the action of acids. In this case, all we are entitled to say is that the immediate surroundings of the soft fibrils differ somewhat in chemical constitution from the parts of the matrix which are more remote; so that under the action of destructive agents the matrix may be split up into sheathing layers round the fibrils and the more solid residuum of the matrix. Miller, in his work, "Micro-Organisms of the Human Mouth," holds to the tubular theory, and says that dentine may be defined as a dense, glue-giving substance, impregnated with lime-salts, and traversed by sheated tubules radiating from the pulp-chamber. They contain living matter, and by means of their many ramifications and anastomoses form a delicate net-work, particularly on the border of the enamel. He has noticed that the sheaths of the tubules are remarkable for their great power of resistance to acids. Black, in writing of dentine, speaks very frequently of the dentinal tubules. Magitot, in 1880, doubts the accuracy of the view ordinarily accepted as to the structure of dentine, denying the existence of any special walls to the tubes (he evidently means the canals), and further argues that it is undesirable to think or speak of the channels as tubes at all. He says they are not tubes in the fresh state, seeing that the fibrils are adherent to the matrix and form a part of it, and that they were originally precisely the same tissue. The existence of sheaths as distinct from the fibrils has also been recently denied by my friend Dr. Sudduth. He says that under the superintendency of the odontoblast, lime-salts are deposited around the rod-like fibrils, and thus form tubular dentine. He repeatedly speaks of tubes in the article which he wrote for "The American System of Dentistry." We quote him as saying that if we hold that distinct and separate dental tubes exist in mature dentine, then we must consider them as having many fine branches, increasing in number as we proceed towards the periphery of the dentine. This, he tells us, is not consistent with our ideas of the character of a tube. No, the nature of dentine is very like that of mature bone. Again he says, the occurrence of interglobular spaces in dentine militates against the tubular theory. The dentinal fibrils pierce the interglobular spaces, and are continuous upon either side; while they make breaks in the continuity of the dentine tissue, yet they do not in any way interfere with the character or form of the dentinal processes. The fact that dentine is not capable of being broken up into tubes is, in his mind, conclusive evidence against the existence of a dentinal sheath, as the wall of a dentinal tube. (Here he evidently means the dentinal canal.) Bödecker, in his recent book, "The Anatomy and Pathology of the Teeth," completely ignores that tissue which investigators for years have called the "sheath of Neumann." In his description of the microscopical appearance of the dentine, he does not use the word "sheath" or "tube" at all. He says, "The dentinal canaliculi are excavations in the basis-substance of the dentine, each containing in its centre a fibre of living matter, and not only the dental canaliculi, but the whole basis-substance of the dentine are pierced by a delicate net-work of living matter."

This is the so-called reticulum which Heitzmann thinks he has found, and which he describes in his bioplasm theory. It is probably the fibrous substructure of connective-tissue which Mummery, of London, recently described, that serves during the forming of the dentine matrix as a scaffolding upon which the gelatinous tissue and the minute spheres of calcoglobulin are deposited. I have described the same appearance in forming enamel in a paper read in Berlin in 1890. I believe this so-called reticulum to be really the scaffolding of connective-tissue fibres, which becomes calcified with the tissue, forming matrix, and not a living reticulum in the fully-formed dentine,—the living matter coming only from the fibrils within the dentinal canals and their branches, which are found throughout the dentine. What are we to understand from the different opinions expressed in the brief review I have given you? What is the definition of the word "tube?" Webster tells us it is "any long and hollow cylinder,—a pipe." A canal he describes as "a duct in the body for the passage of fluid." A duct through which anything is conducted.

If we examine a cross section of a developing tooth, where only a narrow layer of dentine has been formed, we see on the edge of the fully-calcified layer, between it and the formative cells, the transparent, hyaline layer already spoken of. It is somewhat irregular, as if it were formed by the merging of globular masses,—a transitional tissue, which a further stage in the hardening process will completely calcify. It then becomes matrix or basis-substance. It is formed by microscopic globules, calcospherites, which may be seen in the acts of transmigration from the blood-supply of the formative pulp to and within the odontoblasts. These cells appear to superintend the laying of the globules which are arranged in the substance of the gelatinous tissue, a layer of which has been formed by the pulp,—to receive them, they are deposited against the fully-calcified matrix. This is the hyaline layer already spoken of. It is a layer of border-land tissue that is singularly indestructible in acids or in caustic alkalies. I have stated in former papers that there appear to be two kinds of cells concerned in the formation of dentine; one, a fibre-forming cell, with a long process running into the canals; the other, a matrix-forming cell, the true odontoblast. This is usually square and abrupt against the dentine, and the processes, which it appears to have, belong to the fibre cells deeper within the pulp tissue. As the dentine layer forms, the fibre of the fibre-cell lengthens, and against this lengthening fibre this same hyaline layer is formed as against the forming matrix next the formative pulp.

Professor Sudduth tells us that the thickening of the dentinal wall is accomplished by a single layer of odontoblasts which begin the process and persist throughout the life of the pulp. But we frequently see two fibre-cells merged into one, caused by the lessening circumference of the forming dentine; they have merged together, one losing its identity completely at that point. It appears to me clear that all the branching of the canaliculi must be from the merging of these fibre-cells, thus forming branches of the main fibre. The so-called sheath then is found to be a transitional tissue. It is in no sense a separate tissue, and tubes can only be demonstrated after full decalcification when acids *have* completely destroyed the matrix. In cross-sections of the canals in dentine this border-land tissue can be stained by a preparation of nitrate of silver. It acts precisely the same as it does on the hyaline layer of forming dentine. It stains it black. Both tissues are matrix-tissues in a partial state of calcification, and full calcification will take place in this border-land tissue, against the fibre as age comes on, when the dentinal canals are found to be much smaller in diameter than they are in the young tooth. We may assume, then, that the so-called sheath of Neumann is but a transitional tissue only partially calcified, which lines the canals in the dentinal matrix, and is only a tube or sheath when acids have destroyed its adjoining more fully calcified substance.

[The subject-matter of the paper was illustrated by some forty photo-micrographs that had been prepared by Dr. Andrews.]

